# Tissue-Specific RNA-Seq Analysis and Identification of Receptor-Like Proteins Related to Plant Growth in *Capsicum annuum*

**DOI:** 10.3390/plants10050972

**Published:** 2021-05-13

**Authors:** Won-Hee Kang, Boseul Park, Junesung Lee, Seon-In Yeom

**Affiliations:** 1 Institute of Agriculture & Life Science, Gyeongsang National University, Jinju 52828, Korea; wh81kang@gmail.com; 2 Department of Horticulture, Division of Applied Life Science (BK21 Four), Gyeongsang National University, Jinju 52828, Korea; go1004581@naver.com (B.P.); gg6639@naver.com (J.L.)

**Keywords:** *Capsicum* spp., functional analysis, virus-induced gene silencing (VIGS), growth regulation, receptor-like protein (RLP)

## Abstract

Receptor-like proteins (RLPs) are a gene family of cell surface receptors that are involved in plant growth, development, and disease resistance. In a recent study, 438 pepper RLP genes were identified in the *Capsicum annuum* genome (CaRLPs) and determined to be present in response to multiple biotic stresses. To further understand the role of CaRLPs in plant growth and development, we analyzed expression patterns of all CaRLPs from various pepper tissues and developmental stages using RNA-seq. Ten CaRLP genes were selected for further analysis according to transcript levels with hierarchical clustering. The selected CaRLP genes displayed similarity of motifs within the same groups and structures typical of RLPs. To examine RLP function in growth and development, we performed loss-of-function analysis using a virus-induced gene silencing system. Three of the ten tested CaRLPs (CaRLP238, 253, and 360) in silenced plants exhibited phenotypic alteration with growth retardation compared to controls. All three gene-silenced peppers showed significant differences in root dry weight. Only CaRLP238 had significant differences in both root and shoot dry weight. Our results suggest that CaRLPs may play important roles in regulation of plant growth and development as well as function in defense responses to biotic stresses in the RLP gene family.

## 1. Introduction

Plants have cell surface-localized receptors to perceive extracellular signals and communicate with the outside environment. Many plant cell surface receptors have been reported to play key roles in very various processes of plant growth and development [1,2]. There are two major classes of extracellular receptors distinguished by their cytoplasmic domains, namely, receptor-like kinases (RLKs) and receptor-like proteins (RLPs). RLKs contain a single transmembrane domain and a cytoplasmic kinase domain, whereas RLPs lack the cytoplasmic kinase domain and have a short cytoplasmic tail [3].

The major function of an RLP is its well-known defense response against pathogens. The first RLP gene identified, *Cf-9*, was found in tomatoes (*Solanum lycopersicum*) and mediates resistance to the fungal pathogen *Cladosporium fulvum* [4]. Several *Cf* genes in the RLP gene family have been identified from tomatoes [5,6,7,8]. Since then, many RLPs have been identified, and functions implicating disease resistance in plants have been revealed. Tomato *LeEIX2* mediates recognition of the ethylene-inducing xylanase (EIX) of the fungus *Trichoderma viride* [9], tomato *Ve1* also mediates resistance against *Verticillium* [10], and tomato *CuRe1* improves resistance to attack by the plant parasite *Cuscuta reflexa* [11]. The RLP *ReMAX* of Arabidopsis implicates recognition of eMAX from Xanthomonads [12], RFO confers resistance to the vascular wilt fungus *Fusarium oxysporum* forma *specialis matthioli* [13], and RLP23 is also involved in disease resistance [14]. *LepR3* from *Brassica napus* provides race-specific resistance to the fungal pathogen *Leptosphaeria maculans* [15], HcrVf-2 from apple mediates resistance towards the apple scab fungus *Venturia inaequalis* [16], and RLP85 (ELR) from potato confers enhanced resistance to *Phytophthora infestans* [17].

RLPs also play key roles in plant development. In Arabidopsis, *TOO MANY MOUTH* (*TMM*) regulates stomatal distribution on epidermis [18] and is negatively regulated. *CLAVATA2* (*CLV2*) in Arabidopsis is involved in plant biological processes. *CLV2* has a function in maintaining a balanced meristematic stem cell population [19]. *CLV2* is also implicated in the regulation of root apical meristem maintenance. Overexpression of *CLE* genes shows the inhibition of root growth in a *CLV2*-dependent manner [20,21,22,23]. In addition, the *clv2* mutant in Arabidopsis displays novel developmental phenotypes, such as reduced growth, development of more rosette leaves, and shorter stems [24]. In maize, *FASCINATED EAR2* (*FEA2*), an ortholog of *CLV2*, regulates meristem development [25]. Despite these significant insights into the plant growth and development-related function of RLPs, only several RLP genes have been assigned functions in plant development.

Recently, we reported that 438 hot pepper RLP genes in *Capsicum annuum* (CaRLPs) were identified from pepper genomes, and three CaRLPs showed broad-spectrum resistance against variable pathogens [26]. However, plant growth and development-related functions of CaRLPs have not yet been identified. In this study, we conducted expression analysis of 438 CaRLPs using a comprehensive tissue-specific RNA-seq dataset. The 10 CaRLPs with a reads per kilobase per million mapped reads (RPKM) value > 5 of transcripts were selected according to expression profiles and hierarchical clustering. Loss-of-function analysis revealed that three CaRLPs showed phenotypic changes, such as inhibition of root growth, in silenced plants compared to controls. Our study could provide clues for better understanding of potential roles of RLPs in plant growth in pepper and other plants.

## 2. Results

### 2.1. CaRLP Gene Expression Analysis in Various Organs and across Fruit Developmental Stages

**Figure 1:**
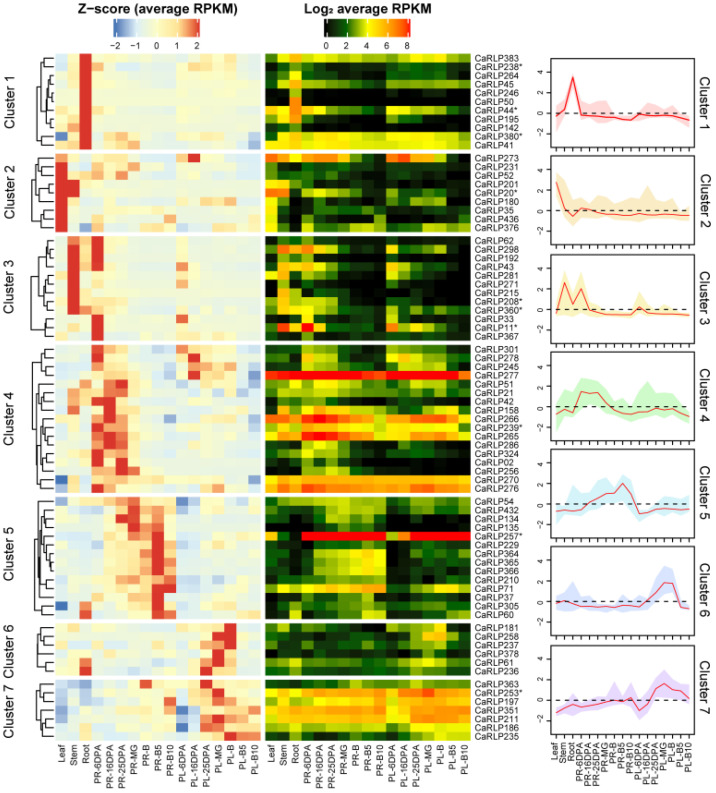
Tissue-specific expression patterns of CaRLPs (*Capsicum annuum* receptor-like proteins). The 76 RLP genes out of 438 genes were selected to construct the heat map. Heatmaps on left and right sides are exhibited using the Z-score and RPKM (reads per kilobase of transcript per million mapped reads), respectively. The selected 10 genes for VIGS (virus-induced gene silencing) assay were marked with asterisks in the right side of the heatmap. The graphs represent the Z-score of CaRLPs in each hierarchical cluster, and red lines indicate median Z-score within a cluster. Key: PR, pericarp; PL, placenta; MG, mature green stage; B, breaker stage; DPA, days post anthesis; B5 and 10, 5 and 10 days post-breaker.

To discover the biological function of CaRLPs in plant growth and development, we obtained the CaRLPs information and tissue-specific RNA-seq data from a previous study [26,27]. Expression analysis of a total of 438 CaRLPs was performed using RNA-seq datasets from the different pepper tissues (root, stem, leaf, pericarp, and placenta), and seven crucial fruit developmental stages. A total of 101 (23.1%) genes out of 438 pepper RLPs were not expressed in any of the five tissues and developmental stages in pepper and were excluded for expression analysis (Supplementary Table S1). We selected CaRLPs with RPKM values of > 5 of transcripts at least in one of all five tissues and developmental stages, and then eventually obtained 76 CaRLPs for further analysis. The expression values of 76 CaRLPs were visualized with Z-score and log_2_RPKM on a heat map (Figure 1). According to the Z-score and RPKM value, 76 CaRLP genes showed various expression patterns, with some CaRLPs evenly expressed in all tested tissues and some genes specifically expressed in only one or a few organs. 

Hierarchical cluster analysis and Z-scores were used to understand the characteristics of 76 CaRLPs. The 76 CaRLPs were clustered into seven expression profiles along with a representative curve displaying each transcript pattern of Z-score (Figure 1). The transcript levels of CaRLPs detected in clusters 1, 2, and 3 were tissue-specific in particular vegetative tissues. Relatively high abundant transcripts in the root, leaf, and stem of CaRLPs clusters were detected compared to other tissues. To determine whether RLP gene members are involved in potential functions during pepper fruit development, we profiled transcripts of pepper RLP genes in the placenta and pericarp at seven crucial stages of fruit development. The CaRLPs in clusters 4 and 5 reached the highest level in early and late pericarp developmental stage, respectively. The 17 CaRLPs in cluster 4 and 14 CaRLPs in cluster 5 may be involved in the placenta developmental stage. In addition, the CaRLPs in clusters 6 and 7 were more highly expressed during the placenta developmental stage than in other tissues, suggesting they may play a role at the reproductive stage. Taken together, these results suggest that CaRLPs might be tissue-specific regulators in vegetative and reproductive tissues in pepper. To validate that the putative functions of CaRLPs expressed in pepper are tissue specific, we analyzed 76 CaRLPs sequences to perform loss-of-function analysis using virus-induced gene silencing (VIGS). Finally, we selected 10 CaRLPs carrying a specific nucleotide sequence in the genome for VIGS study. The information of selected CaRLPs is presented in Table 1.

### 2.2. Structure Analysis of Selected CaRLPs

**Figure 2:**
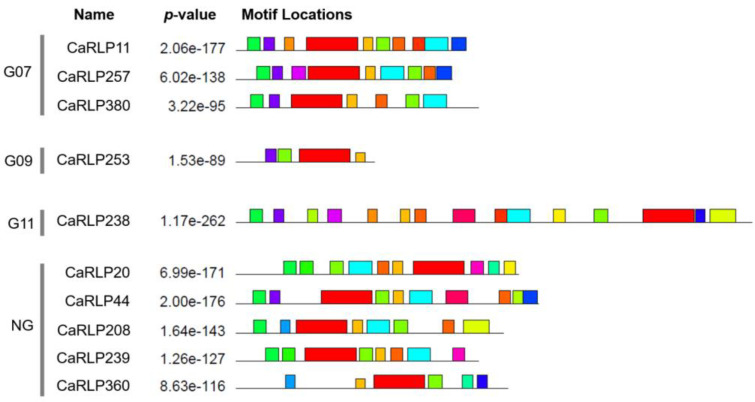
Conserved motif analysis of 10 CaRLPs (*Capsicum annuum* receptor-like proteins). Conserved motifs of the 10 selected CaRLPs were analyzed using the MEME program. The left side of the gene name represents the CaRLP group to which each CaRLP belongs. Each color box in motif locations displays different motifs.

In a previous study, 10 selected CaRLPs were assigned to different pepper RLP groups (Table 1) [26]. Five of the genes were designated as the none-group, three genes constituted Group 07 (G07), one gene was assigned to Group 09 (G09), and one gene was put in Group 11 (G11), which contained the characterized RLP called *CLAVATA2* (*CLV2*). To determine the structure similarities of RLPs, we conducted conserved motifs analyses with 10 CaRLPs. The motif analyses revealed that the 10 CaRLPs had various motif formations according to their group (Figure 2). As predicted, CaRLP11, 257, and 380 in G07 had similar motifs to one other, and there was low similarity of motif composition in the non-group to which five genes belonged. 

**Figure 3:**
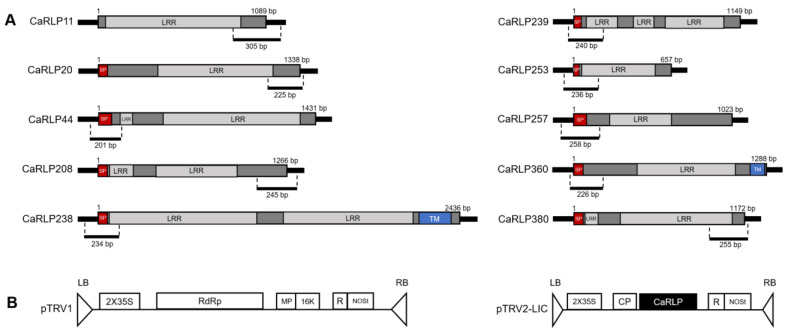
RLP (receptor-like protein) structure and schematic diagram of silenced region of each CaRLP (*Capsicum annuum* receptor-like proteins) and TRV (*Tobacco rattle virus*)-based VIGS (virus-induced gene silencing) vector. (**A**) RLP structure with the diagram of silencing region of CaRLPs. Partial fragment of the gene used for silencing are shown below full CDS (coding sequence) region of each CaRLP. LRR, leucine rich repeat; SP, signal peptide; TM, transmembrane domain. Dark gray region without domain name in CDS indicates non-LRR region. (**B**) Map of the TRV-based VIGS vector used in this study. Partial fragments of each CaRLP were cloned into black colored region (named “CaRLP”) of pTRV2-LIC. LB, left borders of the T-DNA; RB, right borders of the T-DNA; 2X35S, two copies of the *Cauliflower mosaic virus* 35S promoter; CP, coat protein; RdRp, RNA-dependent RNA polymerase; MP, movement protein; 16 K, 16 Kda protein; R, self-cleaving ribozyme; NOSt, nos-terminator.

Based on these motif analyses, the amino acid sequences of 10 CaRLPs were analyzed with the SMART program (http://smart.embl-heidelberg.de/, accessed on 25 March 2021) to predict putative domains and RLP structure. RLPs are cell surface receptors with an extracellular ligand binding domain that is composed of several distinct domains such as a signal peptide, a transmembrane domain, and an extracellular leucine-rich repeat region (LRR) [28]. All 10 CaRLPs contained LRR domains (Figure 3a). Only two CaRLPs (CaRLP238 and 360) had a full-type structure with three functional domains, such as signal peptide, transmembrane domains, and LRRs. Seven genes contained the two domains including signal peptide and LRRs. The remaining one gene (CaRLP11) had only a single domain with LRRs (partial-type RLP). Taken together, the selected CaRLP genes displayed similarity of motifs within the same groups and showed typical structures of RLPs.

### 2.3. Phenotypic Alterations in Root Growth of CaRLP-Silenced Plants

**Figure 4:**
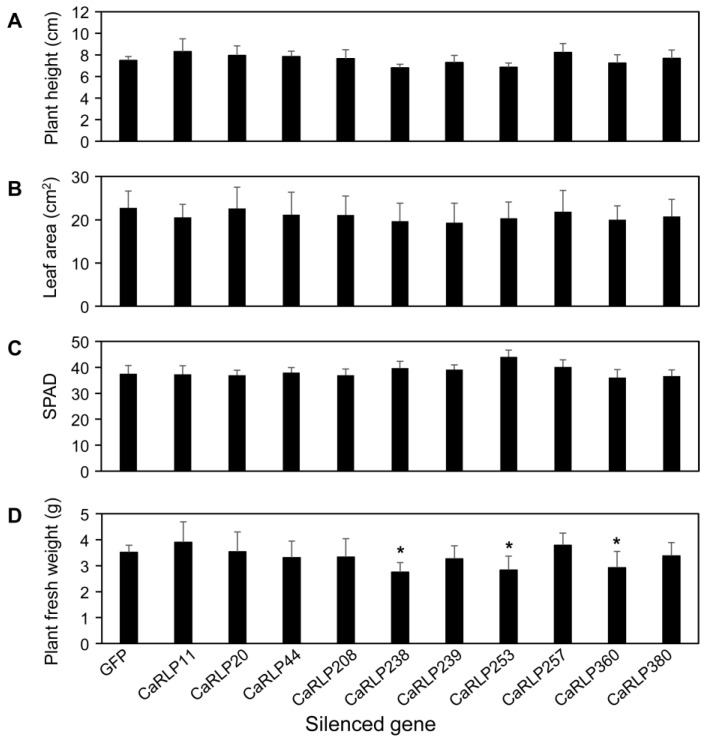
Phenotype analysis of 10 CaRLP (*Capsicum annuum* receptor-like proteins)-silenced pepper plants. Comparison of plant height (**A**), leaf area (**B**), chlorophyll contents (**C**), and fresh weight (**D**) in control plants (GFP; green fluorescent protein) and CaRLP-silenced plants. CaRLP-silenced plants and control plants were used for phenotypic analysis at six weeks after silencing. The plant height was measured from the ground to the growth point of the shoot. The plant fresh weight included shoot and root weight. The leaf area and chlorophyll contents were measured on the third true leaf and third and fourth leaves, respectively. Data are shown as mean ± SD. Statistically significant differences compared with the TRV2-GFP control determined using Student’s t-test are indicated by asterisks (* *p* < 0.05).

To investigate the possible functions of CaRLPs in the development of peppers, 10 CaRLP genes were silenced using TRV-mediated VIGS (Figure 3b). Approximately, 200–300 bp cDNA fragments carrying the gene-specific sequences of each CaRLP gene were inserted in the TRV2 vector for the loss-of-function study (Figure 3a,b). We monitored plant growth and morphological changes, including plant height, chlorophyll contents, leaf area, and plant fresh weight, for six weeks after silencing. Even though silenced plants exhibited slight differences in the aforementioned phenotypes compared to control plants (TRV-GFP), eventually there were no significant differences in plant height, leaf area, and chlorophyll contents in CaRLP-silenced plants compare to controls (Figure 4a–c). However, plant fresh weight of three CaRLP-silenced plants (CaRLP238, CaRLP253, and CaRLP360) were significantly reduced compared to that of control plants. The level of fresh weight in these three CaRLP-silenced plants was decreased 0.7–0.85-fold compared with control plants (Figure 4d). These results indicate that the three CaRLP genes may be involved in plant biological processes in peppers. 

**Figure 5:**
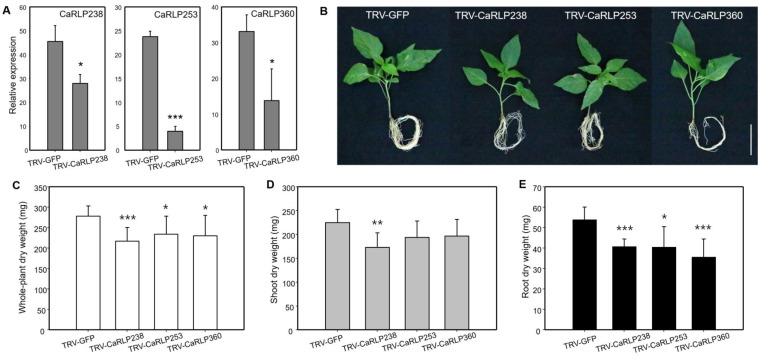
Phenotypic characterization of CaRLP238, 253, and 360-silenced plants. (**A**) Semi-quantitative reverse transcription-polymerase chain reaction (Semi-quantitative RT-PCR) analyses of the each CaRLP (*Capsicum annuum* receptor-like proteins) in silenced pepper leaves. TRV-GFP (*Tobacco rattle virus*-green fluorescent protein)-treated mock plants were used for VIGS as a reference sample. Expression values were normalized to levels of *CaActin* gene expression. Data represent mean ± SD (Student’s t-test, * *p* < 0.05, ** *p* < 0.01, *** *p* < 0.0001). (**B**) Phenotype of CaRLP238, 253, and 360-silenced plant and control plants (TRV-GFP). The photo was taken at six weeks after agro-infiltration for a VIGS assay. Scale bar indicates 5 cm. (**C**–**E**) Comparison of whole-plant dry weight, shoot dry weight, and root dry weight in control plant (TRV-GFP), and CaRLP238, 253, and 360 silenced plants. Data are shown as mean ± SD. Statistically significant differences compared with the TRV2-GFP control determined using Student’s t-test are indicated by asterisks (* *p* < 0.05, ** *p* < 0.01, *** *p* < 0.0001).

To further understand a possible function, we observed the silencing effects of three CaRLPs (CaRLP238, CaRLP253, and CaRLP360) in the growth of shoots and roots. The expression level of each of the three CaRLPs, determined using semi-quantitative RT-PCR, was significantly lower in CaRLP-silenced plants than in the TRV2-GFP control plants (Figure 5a). Slight phenotypic alterations among the three CaRLP-silenced plants and TRV-GFP plants were observed. (Figure 5b). Even though significant differences were not detected in the plant height of CaRLP-silenced plants compared with control plants (Figure 3a), the growth of plant shoots in CaRLP-silenced plants was smaller than that of control plants (Figure 5b). The root phenotype of three CaRLP-silenced plants showed inhibition of root growth compared with control plants. In addition, we measured the dry weight of the whole plant, shoot, and root in three CaRLP-silenced plants and control plants. The whole-plant dry weight was significantly reduced in all three CaRLP-silenced plants compared to controls (Figure 5c). Interestingly, only CaRLP238-silenced plants had significantly decreased shoot dry weight compared with controls (Figure 5d). Root dry weights of all three CaRLP-silenced plants were significantly reduced compared to controls. Taken together, suppression of three genes resulted in retardation of shoot and/or root growth, suggesting that these genes may be involved in regulation of plant normal growth.

## 3. Discussion

The RLP gene family is one of the largest super families involved in various biological functions in plants, yet only a few RLP genes have been functionally characterized regarding biological processes. The RNA-seq has been widely applied to research including the analysis of gene expression and prediction of gene function since next-generation sequencing has been easily available to use. Several studies have reported the genome-wide identification of RLP genes in Arabidopsis, rice, tomato, Brassica, and legumes [24,29,30,31,32,33]. However, no previous study has reported the genome-wide transcriptional profiles in organ-specific of RLPs in pepper. Previously, we identified 438 pepper RLP genes and a comparative transcriptomic analysis revealed the role of core RLP regulators in disease resistance against various pathogens [26]. In the study reported here, we attempted to discover the additional function involved in plant growth by analyzing pepper tissue-specific transcriptome. A total of 76 CaRLPs had variable expression patterns whereby some were expressed evenly in all stages tested or specifically in one or a few stages (Supplementary Table S1). The hierarchical clustering of 76 CaRLPs was detected via categorization into seven clusters showing distinct tissue-specific expression patterns. Among them, ten CaRLP genes having specific sequences and distinguishable expressions were selected for further analysis. 

Motif analysis of 10 CaRLP proteins revealed that CaRLPs had 20 motifs in total and shared conserved motifs in the same subgroup, which is consistent with a previous study [26]. The distribution of motifs also showed the conserved position of each gene in the same group (Figure 2). This result suggested that the genes in the same group might have similar evolutionary histories and possibly a similar function [34,35]. RLPs are pattern recognition receptors containing N-terminal signal peptides, transmembrane domains, and LRR [28]. However, some proteins without transmembrane domains or without signal peptides are considered as RLPs [29,31]. A previous study reported RLPs without a transmembrane domain attached to the extracellular side of the plasma membrane using a glycosylphosphatidylinositol (GPI) anchor [36]. In the case of the tomato, structure analysis of tomato RLPs show that 30.7% of genes of all tomato RLPs are considered full-type RLPs encompassing all three domains and 42.6% of genes contain two domains [30]. Therefore, even though some CaRLPs were predicted as partial-type RLPs (not having all three domains) in this study, they could be considered as a typical RLP gene.

Most biological processing genes are under purifying selection to maintain specific functions [37]. Therefore, developmental genes are less likely to be duplicated compared to gene families for stress adaptation, like resistance genes, and they are more structurally and functionally conserved among plant species. Moreover, analysis of Arabidopsis and rice RLP genes shows that developmental genes are less likely to be duplicated and undergo diversifying selection than disease-resistance genes [24]. For instance, *CLV2* is involved in plant growth and development processes, including shoot meristem development, root apical meristem maintenance, and organ development [2,19]. The *FASCIATED EAR2* (*FEA2*), an orthologue of *CLV2* involved in meristem development, shows a similar mutant phenotype to *CLV2* [25]. Previously, a small number of CaRLP genes were classified with *TMM* and *CLV2* as Group 10 and 11, respectively. The CaRLPs in Group 10 and 11 were predicted to duplicate before tomato/pepper speciation, suggesting evolutionary pressure to conserve function [26]. Interestingly, CaRLP238, which shows most phenotypic changes in both root and shoot of silenced pepper plants in this study, was grouped with *CLV2* in Group 10 (Table 1) based on a phylogenetic tree of sequence similarity. According to the aforementioned results, it can be predicted that genes belonging to groups like *CLV2* and *TMM* could possibly function in relation to plant development. Therefore, functional analysis of other CaRLPs belonging to the same group as *CLV2* and TMM, through further research, might provide clues to find biological roles in plant biological processes for the RLP gene family.

To understand their role in plant growth, 10 selected genes were silenced in pepper plants, and then three CaRLP genes (CaRLP238, CaRLP253, and CaRLP360) were characterized further. CaRLP238, highly expressed in the root (Figure 1), displayed morphological changes in growth after silencing such as reduced dry weight of whole plant, shoot, and root. Additionally, CaRLP253 and 360 displayed high expression levels in placenta and stem, respectively, but they showed significant differences in root dry weight when silenced. For these three CaRLP genes, although additional phenotypic analysis of other tissues was not performed, these CaRLPs might be involved in growth processes in peppers. However, in order to unveil the function, further studies including more morphological analysis and molecular biology analysis during growth and fruit development will be needed. 

Although the expression level of silenced genes was significantly lower than control plants, one gene, CaRLP238, may not be down-regulated enough to compromise CaRLP function (Figure 5a). A VIGS assay could be a very useful tool for assessing gene functions and this tool has been developed in a wide variety of plant species [38]. However, VIGS has weaknesses, including partial silencing, that require consideration and prudence in its use. Partial silencing happens in an unpredictable manner. Therefore, less obvious phenotypes could impair the use of VIGS in the analysis. This problem can be addressed by increasing the sample size to include enough individuals [39,40]. In this study, we performed more than three independent assays for the VIGS that consisted of at least eight to ten plants for a replicate, totally more than thirty plants. In the case of silencing reduction, even though the silencing efficiency of CaRLP238 genes was approximately 40% lower compared to the control, we achieved the same stable results and phenotypic alteration-like significant differences in whole plant weights including both shoot and root compared to controls. In addition, a previous report showed that using VIGS, even target genes were reduced between 65 and 24% (*p* < 0.05) compared to WT plants and the silenced plants also exhibited clear phenotypic alterations [41]. Taken together, three CaRLPs (CaRLP238, 253, and 360) showed acceptable and reasonable ranges in expression reduction to phenotypic changes.

## 4. Materials and Methods

### 4.1. CaRLP Information, RNA-Seq Data, and Expression Profiling

We obtained gene information and sequences of CaRLP genes from a previous study [26]. The RNA-seq of five pepper tissues, including root, stem, leaf, pericarp, and placenta [42], were used for the expression analysis of CaRLPs. The pericarp and placenta RNA-seq contained seven developmental stages: 6, 16, and 25 days post anthesis; mature green (MG); breaker (B); and 5 and 10 days post breaker. All transcriptome data were analyzed using in-house pipelines [43,44]. The expression values of tissues were normalized using RPKM. To analyze expression profiles of CaRLPs, CaRLPs with RPKM values of >5 at least in one of all five tissues and developmental stages selected, and then, 76 CaRLPs were obtained. The expression patterns and hierarchical clustering of significant genes were visualized with Z-score and log_2_ RPKM using Heatmap by R package (http://bioconductor.org/, accessed on 25 March 2021). Of 76 CaRLPs, we selected 10 CaRLPs possessing a gene-specific sequence for further analysis.

### 4.2. Motif Analysis and Structure Analysis of CaRLP Genes

Identification of conserved motifs in 10 selected CaRLPs was performed using the MEME suite (http://meme-suite.org/tools/meme, accessed on 25 March 2021). The analysis parameters were as follows: maximum number of motifs, 20; minimum width of motifs, 15; maximum width of motifs, 200, and other details were used with default settings. To find functional domains of RLPs, 10 selected CaRLPs were analyzed using functional structure annotation with SMART (http://smart.embl-heidelberg.de/, accessed on 25 March 2021) with default values.

### 4.3. Gene Cloning for Virus-Induced Gene Silencing (VIGS) Assay

*Capsicum annuum* (‘Nockwang’) was used in this study for functional analysis. Seedlings were grown in a growth chamber at 25 °C under a 16 h light/8 h darkness cycle. The newly emerging cotyledons of germinating pepper seedlings were used for a VIGS assay.

To construct the plasmids for analysis of loss-of-function, *Tobacco rattle virus* (TRV)-based VIGS vectors containing pTRV1 and pTRV2-LIC were used. To find gene-specific sequences of CaRLPs for VIGS, 10 CaRLPs performed BLASTn against the *C. annuum* ‘CM334’ v. 1.55 genome. The gene-specific region, which is the 3′ or 5′ CDS region with untranslated region (UTR), of each CaRLP was amplified using Solg^TM^ Pfu-X DNA polymerase (Solgent, Daejeon, Korea) with primers (Supplementary Table S2). The PCR products were cloned into the pTRV2-LIC vector according to a method used in a previous study [45,46]. The pTRV2-LIC-CaRLP plasmids were transformed into *Agrobacterium tumefaciens* strain GV3101 using the freeze-thaw method. *Agrobacterium* cultures, including pTRV1 and pTRV2-LIC-CaRLP, were mixed in a 1:1 ratio (OD_600_ = 0.5) and infiltrated into two fully expanded cotyledons of pepper seedlings (approximately 10 days after germination) using a needleless 1-mL syringe. In this experiment, TRV2-green fluorescent protein (GFP) and TRV2- phytoene desaturase (PDS) were used as a negative and positive control for VIGS, respectively.

### 4.4. Semi-Quantitative RT-PCR Analysis

The total RNA was extracted from pepper leaves using the TRIzol reagent (Thermo Fisher Scientific, Inc., Waltham, MA, USA) according to the manufacturer’s instructions. cDNA was synthesized from 2 µg of total RNA using oligo d(T) primer and M-MLV reverse transcriptase (Promega, Madison, WI, USA) following the manufacturer’s protocol. Each gene-specific primer was designed to detect the levels of silenced-CaRLP gene expression. For semi-quantitative RT-PCR reaction, we tested the RT-PCR cycles between 26–32 cycles with all samples to ensure the linearity of the results. Then, to determine efficiency of gene silencing, semi-quantitative RT-PCR was performed using rTaq polymerase (Takara, Shiga, Japan) on a MiniAmp™ Thermal Cycler (Thermo Fisher Scientific Inc., Waltham, MA, USA) under the following conditions: initial denaturation step was 94 °C for 2 min, followed by 30 cycles of 94 °C for 30 s, then an annealing step at 58 °C for 30 s, and an elongation step at 72 °C for 30 s. The pepper actin gene (*CaActin*) [47] was used as reference gene. All PCR reactions were repeated in triplicate using at least three independent samples. To determine quantification of RT-PCR products, images of the amplicon in ethidium bromide-stained agarose gel were obtained using WiseCaptureII software (Daihan Scientific Co., Ltd., Wonju, Korea). Quantification of the amplicon bands in agarose gel was measured using ImageJ software (https://imagej.nih.gov/ij/, accessed on 25 March 2021). Band intensity of *CaActin* of each cDNA sample was calculated to normalize for variation in sample concentration. Mean and standard deviation of each CaRLP expression level in silenced pepper plants and mock plants (TRV-GFP) were calculated after normalization to *CaActin*. Statistically significant differences (*p* < 0.05) of CaRLP expression levels in CaRLP-silenced plants were verified by comparison with those of the control plant (TRV-GFP). It was determined using one-way analysis of variance (ANOVA) and Student’s t-test using SAS software (version 9.4; SAS Inc., Cary, NC, USA).

### 4.5. Phenotypic Analysis of CaRLP-Silenced Pepper Plants

The morphological changes of each CaRLP-silenced pepper plant were measured, such as plant height (from soil surface to shoot growing point, cm), leaf area (3rd true leaf, cm^2^), plant fresh weight of whole plant (mg), and chlorophyll contents (SPAD value) at 6 weeks after silencing. Relative chlorophyll contents of the 3rd and 4th leaves of CaRLP-silenced plants were measured with a Chlorophyll Meter (Minolta Camera Co., Ltd., Japan). The silenced plants at 6 weeks after a VIGS assay were dried at 65 °C in an oven for two days, and then, dry weights of whole plants, shoot, and root were measured. All phenotypic analyses were performed using more than three independent experiments, with 8–10 plants for each experiment. Statistically significant differences (*p* < 0.05) of phenotypic analysis between control plants (TRV-GFP) and CaRLP-silenced plants (TRV-CaRLP) were determined using one-way ANOVA and Student’s t-test using SAS software (version 9.4; SAS Inc., Cary, NC, USA).

## Figures and Tables

**Table 1:** Information for VIGS (virus-induced gene silencing) analysis of 10 selected CaRLPs (*Capsicum annuum* receptor-like proteins).

CaRLP ID	Location	CDS Size (bp)	Protein Size (aa)	Group
CaRLP11	Ch1	1089	362	G07
CaRLP20	Ch2	1338	445	NG
CaRLP44	Ch2	1431	476	NG
CaRLP208	Ch7	1266	421	NG
CaRLP238	Ch8	2436	811	G11
CaRLP239	Ch8	1149	382	NG
CaRLP253	Ch8	657	218	G09
CaRLP257	Ch9	1023	340	G07
CaRLP360	Ch0 ^1^	1288	428	NG
CaRLP380	Ch0	1172	382	G07

^1^ Ch0 indicates unmapped scaffolds on chromosome.

## Data Availability

The data supporting the findings of this study are available within the article and its supplementary materials.

## Supplementary Materials

The following are available online at https://www.mdpi.com/article/10.3390/plants10050972/s1, Table S1: Reads per kilobase of transcript per million mapped reads (RPKM) from tissue-specific RNA-seq of pepper RLP genes, Table S2: Primers used for VIGS (virus-induced gene silencing) analysis.
